# Following the World Health Organization’s Recommendation of Exclusive Breastfeeding to 6 Months of Age Does Not Impact the Growth of Rural Gambian Infants^1^,^2^,^3^

**DOI:** 10.3945/jn.116.241737

**Published:** 2016-12-21

**Authors:** Kamilla G Eriksen, William Johnson, Bakary Sonko, Andrew M Prentice, Momodou K Darboe, Sophie E Moore

**Affiliations:** 4 Medical Research Council (MRC) Elsie Widdowson Laboratory, Cambridge, United Kingdom;; 5 MRC Unit The Gambia, Banjul, The Gambia;; 6 MRC International Nutrition Group, London School of Hygiene and Tropical Medicine, London, United Kingdom; and; 7 Division of Women’s Health, King’s College London, London, United Kingdom

**Keywords:** exclusive breastfeeding, infant feeding practice, postnatal growth, The Gambia, multilevel modelling, reverse causality

## Abstract

**Background:** The WHO recommends exclusive breastfeeding (EBF) for the first 6 mo of life.

**Objective:** The objective of this study was to assess the benefit of EBF to age 6 mo on growth in a large sample of rural Gambian infants at high risk of undernutrition.

**Methods:** Infants with growth monitoring from birth to 2 y of age (*n* = 756) from the ENID (Early Nutrition and Immune Development) trial were categorized as exclusively breastfed if only breast milk and no other liquids or foods were given. EBF status was entered into confounder-adjusted multilevel models to test associations with growth trajectories by using >11,000 weight-for-age (WAZ), length-for-age (LAZ), and weight-for-length (WLZ) *z* score observations.

**Results:** Thirty-two percent of infants were exclusively breastfed to age 6 mo. The mean age of discontinuation of EBF was 5.2 mo, and growth faltering started at ∼3.5 mo of age. Some evidence for a difference in WAZ and WHZ was found between infants who were exclusively breastfed to age 6 mo (EBF-6) and those who were not (nEBF-6), at 6 and 12 mo of age, with EBF-6 children having a higher mean *z* score. The differences in *z* scores between the 2 groups were small in magnitude (at 6 mo of age: 0.147 WAZ; 95% CI: −0.001, 0.293 WAZ; 0.189 WHZ; 95% CI: 0.038, 0.341 WHZ). No evidence for a difference between EBF-6 and nEBF-6 infants was observed for LAZ at any time point (6, 12, and 24 mo of age). Furthermore, a higher mean WLZ at 3 mo of age was associated with a subsequent higher mean age at discontinuation of EBF, which implied reverse causality in this setting (coefficient: 0.060; 95% CI: 0.008, 0.120).

**Conclusion:** This study suggests that EBF to age 6 mo has limited benefit to the growth of rural Gambian infants. This trial was registered at http://www.isrctn.com as ISRCTN49285450.

## Introduction

The WHO recommends exclusive breastfeeding (EBF)^8^ for the first 6 mo of an infant’s life, with continued breastfeeding up to 2 y of age or beyond, along with nutritionally adequate, safe, and appropriate complementary foods (1). Optimal breastfeeding practices have been shown to have clear short-term advantages for child morbidity and mortality, especially in low- and middle-income countries (LMICs) (2). A systematic review that supports the EBF recommendation analyzed several studies from low-, middle-, and high-income countries and reported that EBF to 6 mo of age compared with EBF to 3–4 mo of age with continued mixed breastfeeding (introduction of complementary liquids or solid foods) resulted in lower morbidity from gastrointestinal infection and prolonged lactational amenorrhea (3).

In many LMICs, infants are small at birth, show catch-up growth in the first few months of life, and then enter a period of reduced growth velocity, which results in substantial growth faltering by the second year of life (4, 5). The growth faltering usually begins within the first 6 mo of life (4–7). However, there is limited evidence as to how EBF to 6 mo affects infant growth. The majority of studies were conducted in affluent countries or urban areas in LMICs, where overweight and obesity is a greater problem than growth faltering and where nonexclusively breastfed children are often or usually given infant formula (8–12). A growing number of trials that randomly assigned mothers to EBF counseling have been implemented in low-income settings (13–17); however, only 2 trials investigated the effect that EBF to age 6 mo has on growth in settings in which no infant formula was consumed (13, 14). In these 2 studies, both conducted in Honduras, there was no difference in weight or length at 6 mo of age between infants who were exclusively breastfed to 6 mo compared with to 4 mo (with continued breastfeeding and solid foods). These studies were, however, criticized for having a low sample size (*n* = 119 and *n* = 97, respectively) (3), and only 1 of these trials followed the infants to 1 y of age (13). Observational studies from LMICs have also investigated this topic and found similar results, that EBF to 6 mo has limited benefit to growth (18, 19). However, the majority of the observational studies were either cross-sectional or longitudinal studies that analyzed serial measurements as cross-sectional data, which reduces the power of longitudinal data (20).

The Gambia is a low-income country in West Africa, where food availability and nutritional status in rural areas are poor. A large proportion of children in this setting experience substantial growth faltering (5) and women are at great risk of several micronutrient deficiencies (21). Furthermore, in rural areas, food availability and nutritional status are strongly influenced by seasonality, and a chronically marginal diet is exacerbated by a “hungry season,” when food stocks from the previous harvest season are depleted (22). In this analysis, we used longitudinal data on growth and infant feeding practices for 756 infants from rural Gambia to investigate whether following the WHO EBF recommendation is associated with better growth from birth to 2 y of age.

## Methods

### 

#### Study population.

**FIGURE 1:**
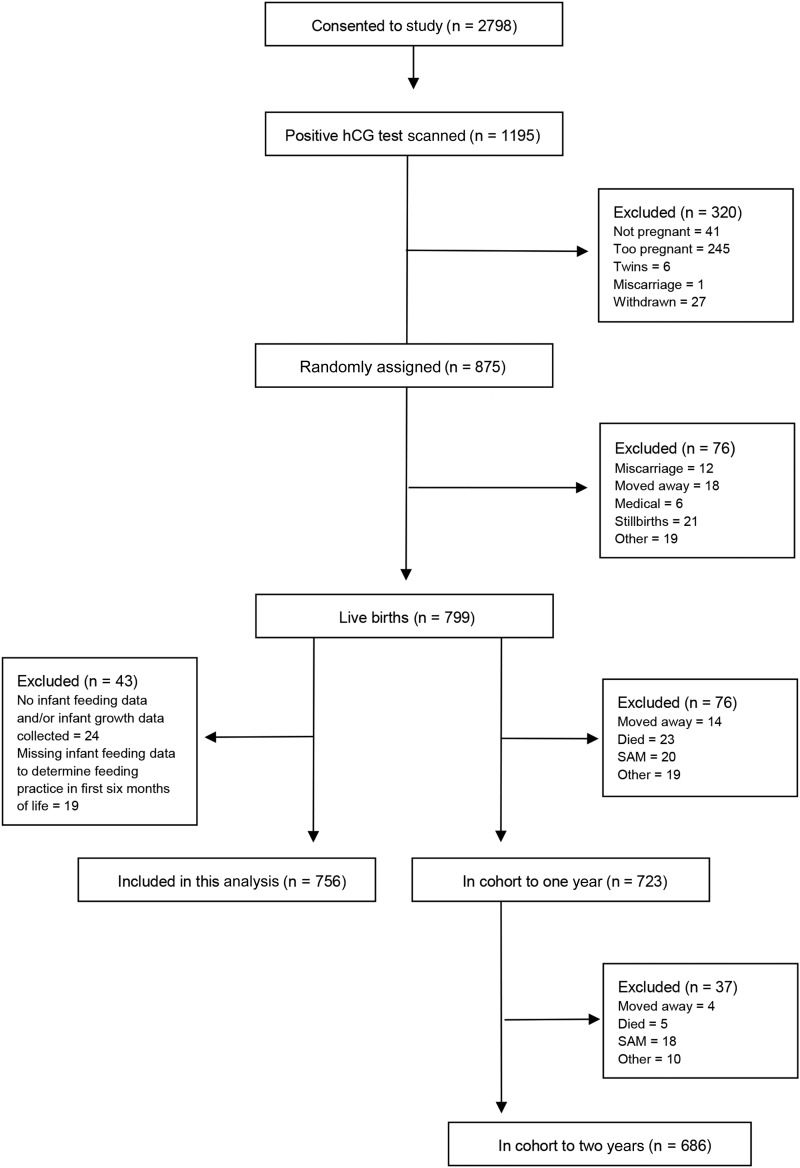
Flow diagram of included and excluded participants in the ENID trial and in this analysis. ENID, Early Nutrition and Immune Development; hCG, human chorionic gonadotropin; SAM, severe acute malnutrition.

The current analysis used data collected as part of the Early Nutrition and Immune Development (ENID) Study, a randomized trial conducted in the West Kiang region of The Gambia between April 2010 and February 2015. The current post hoc analysis was not planned in the original study design. Full details of the main ENID trial can be found in the published trial protocol (23). The main trial followed pregnant women and their infants to 1 y of age; however, here we additionally used data from the ENID-Growth Study, which is an extension of the main ENID trial, in which follow-up of infants was continued to 2 y of age. Briefly, women of reproductive age (18–45 y) were recruited to assess the effect of combined prenatal and infant nutritional supplementation on infant immune development. Pregnant women were randomly assigned, in a partially blinded fashion, to a supplement group when they booked for antenatal care (before 20 wk of gestation), with supplementation continuing until delivery. Nurses, midwives, and field- and community health workers were trained in optimal breastfeeding practices; however, no counseling to the participating women was implemented beyond what is standard practice in this baby-friendly community. The women were randomly assigned to one of the following intervention arms: *1*) iron + folic acid, *2*) multiple micronutrients, *3*) protein-energy and iron+folate, or *4*) protein-energy and multiple micronutrients. Their infants were further randomly assigned from 6 to 18 mo of age to a supplement group of lipid-based nutritional supplements fortified with multiple micronutrients or to a group of lipid-based nutritional supplements and followed up to 2 y of age. A total of 2798 women consented to the ENID study, and of these, 875 were eligible for supplementation. The infants were born between August 2010 and February 2014, and 756 infants were included in this analysis (**Figure 1**).

#### Anthropometric measurements.

Infants had anthropometric measurements taken at birth (within 72 h of delivery) and at scheduled visits to the Medical Research Council (MRC) Keneba field station at 1, 8, 12, 24, 52, 78, and 104 wk of age, with additional home visits at 16, 20, 32, 40, 65, and 91 wk by trained fieldworkers. An embedded substudy measured ∼200 infants at the additional time points of 4, 28, 36, 44, and 48 wk of age with the use of the same procedures and anthropometric equipment as the main follow-up (see **Supplemental Table 1** for data availability according to time point). All weights and lengths were measured by using electronic scales and length boards, which were precise to 10 g and to 1 mm, respectively. Fixed-length boards (Seca 417) were used for all clinic and field visits after the neonatal home visit, for which a flexible-length mat was used. Weight and length measurements were converted to weight-for-age *z* score (WAZ), length-for-age *z* score (LAZ), and weight-for-length *z* score (WLZ) according to the WHO growth standards by using the WHO Anthro program (version 3.2.2; January 2011). The data set consisted of >11,100 assessments of WAZ, LAZ, and WLZ, with a mean of 14.8 assessments/infant (range: 2–19 assessments) over a mean of 23 mo (range: 0.2–25 mo).

#### Infant feeding practice and morbidity data.

Trained fieldworkers collected weekly infant feeding and morbidity data by questionnaire at home visits. At these visits, the mother or caregiver was asked to recall infant feeding practices in the previous 7 d (i.e., if the infant was breastfed, and if other foods or drinks had been introduced, and the frequency of these other foods and/or drinks). The mother or caregiver was further asked if the child had experienced any diarrhea, vomiting, cough, rapid breathing, or fever in the past 7 d.

#### Exposure and confounding variables.

Infant feeding practice was defined as being exclusively breastfed to age 6 mo (EBF-6; provision of breast milk only) compared with not being exclusively breastfed to age 6 mo (nEBF-6). The nEBF-6 infants were either predominantly breastfed (provision of breast milk and liquids only) or partially breastfed (provision of breast milk and solid foods) in the first 6 mo.

The following variables were investigated as potential confounders or effect modifiers: maternal age, parity, weight (measured in the first trimester of pregnancy), height, BMI, educational level (completed years of either English or Arabic schooling), supplementation group during pregnancy, village, gestational age at birth, and incidence of infant diarrhea (defined as having ≥3 loose stools/d) and morbidity (defined as combined episodes of diarrhea, vomiting, cough, rapid breathing, or fever).

“Village” was defined as participants from 1 of the 4 “core” villages of Jali, Kantong Kunda, Keneba, and Manduar compared with participants from 1 of the remaining 24 villages. This division was chosen because the core villages are situated close to the MRC Keneba field station and therefore are in closest proximity to the MRC Keneba clinic, which has a known influence on health-seeking behavior (24).

#### Statistical analysis.

The crude association between infant feeding practice and continuous data was investigated by using *t* tests; for categorical data, chi-square tests were used. Multilevel models (MLMs) were used for the analysis of longitudinal data. Individual age-related trajectories for each of WAZ, LAZ, and WLZ were modeled separately in an MLM with measurement occasion at level 1; individuals at level 2; incorporating infant feeding practice associations with the sample-mean growth trajectories and adjustment for confounders and competing effects.

The shape of each trajectory was specified as a restricted cubic spline, with 4 knots (0.008, 0.389, 0.797, and 1.993 y). This model was chosen because it provided the lowest deviance compared with quadratic, cubic, and fractional polynomial growth-curve models. The constant and the cubic spline age terms were allowed to have a random effect at level 2, allowing deviations from the intercept and gradient of the mean trajectory for each infant. Infant feeding practice was entered as follows: *1*) as a main effect, representing the association of infant feeding practice with the outcome at the intercept (i.e., at birth), and *2*) as an interaction with the spline age terms, representing its association with the slope or rate of change in WAZ, LAZ, and WLZ. We used an unstructured variance-covariance matrix for the level 2 random effects.

Seasonality of infant anthropometric measurements was included in the MLMs with the use of Fourier’s term (25). The Fourier series is a statistical model that allows the decomposition of any periodic function into a linear combination of simple oscillating functions (sines and cosines) parameterized by coefficients (the Fourier coefficients) (25). In this analysis, the first 4 sets of Fourier terms were used. Infant morbidity incidence was added to the model as a time-dependent variable, and the rest of the potential confounding and competing effect variables were added as time-independent variables (for the full-model equation, see the **Supplemental Appendix**).

Overall model fits were improved by removing all length measurements at birth because residuals were large, assumedly due to high measurement error. Birth measurements of length were often taken in the subject’s home, and the use of a flexible-length mat likely introduced error. Data with large level 1 residuals (defined as >2 or <−2 *z* scores) were removed because they were outliers and assumed to reflect large measurement error (for WAZ, LAZ, and WLZ, *n* = 8, 28, and 71 data points were removed, respectively). The final MLMs had residual SDs of 0.38, 0.49, and 0.58 *z* scores for WAZ, LAZ, and WLZ, respectively, which indicates the overall goodness-of-fit of the models. As shown, the WLZ data did not fit the cubic spline model as well as LAZ and WAZ, however as WLZ includes 2 sources of measurement errors (in weight and length), the residual SD is inevitably larger for this model compared to WAZ and LAZ (20).

The fully adjusted models were used to estimate between–infant feeding group differences at selected infant ages (0, 6, 12, and 24 mo), which are presented with 95% CIs. Furthermore, trajectories were plotted according to infant feeding practice. All of the statistical analyses were performed with STATA version 14 (StataCorp).

#### Ethics, governance, and trial registration.

The ENID and ENID-Growth trials were approved by the joint Gambia Government/MRC Unit, The Gambia Ethics Committee (projects SCC1126v2 and L2010.77, respectively). Written informed consent was obtained from all of the women before enrollment into the trial. The trial observed Good Clinical Practice Standards and the current version of the Helsinki Declaration. The ENID trial was registered as ISRCTN49285450.

## Results

### 

#### Maternal and infant characteristics.

A total of 756 mothers were included in the analysis, with a mean ± SD age of 30.3 ± 6.9 y (**Table 1**). The population was lean: 19% of the mothers were underweight [BMI (in kg/m^2^) <18.5] and only 11% were either overweight or obese (BMI ≥25) at enrollment into the study (mean gestational age at enrollment: 13.7 wk). Educational levels were low, with 78% of women having received no formal education. The 756 infants included in this analysis were born with a mean ± SD birth weight of 3.017 ± 0.4 kg, 8% had low birth weights (<2.5 kg), and 22% were small for gestational age. In the first 2 y of life, the mean number of episodes of diarrhea and other morbidity were 4.3 ± 3.5 and 16.4 ± 8.4, respectively.

**TABLE 1:** Description of study population according to infant feeding practice^1^

Variable	*n*	Exclusively breastfed to age 6 mo	Not exclusively breastfed to age 6 mo	All
Maternal age, y	755	29.9 ± 6.4	30.4 ± 6.9	30.3 ± 6.9
Maternal weight, kg	755	55.3 ± 9.4	55.7 ± 9.6	55.6 ± 9.5
Maternal height, cm	756	161.7 ± 5.7	162.1 ± 5.9	162.0 ± 5.8
Maternal BMI, kg/m^2^	755	21.2 ± 3.6	21.2 ± 3.3	21.2 ± 3.4
Parity categories, *n* (%)				
Primiparous	88	32 (13.5)	56 (10.8)	88 (11.6)
Multiparous	668	206 (86.5)	462 (89.2)	668 (88.4)
Maternal education categories, *n* (%)				
No education	591	192 (80.7)	399 (77.0)	591 (78.2)
Low education (1–7 y)	94	25 (10.5)	69 (13.3)	94 (12.4)
Medium education (8–14 y)	71	21 (8.8)	50 (9.7)	71 (9.4)
Maternal supplementation categories, *n* (%)				
Fe-Fol	191	66 (27.7)	125 (24.1)	191 (25.3)
MMNs	194	55 (23.1)	139 (26.8)	194 (25.7)
PE + Fe-Fol	179	63 (26.5)	116 (22.4)	179 (23.7)
PE + MMNs	192	54 (22.7)	138 (26.6)	192 (25.4)
Birth weight, kg	629	3.045 ± 0.4	3.005 ± 0.4	3.017 ± 0.4
Birth-weight categories, *n* (%)				
Low (<2.5 kg)	53	14 (7.2)	39 (9.0)	53 (8.4)
Normal (2.5–3.9 kg)	572	179 (91.8)	393 (90.6)	572 (90.9)
High (≥4.0 kg)	4	2 (1.0)	2 (0.5)	4 (0.6)
Birth length, cm	751	50.5 ± 2.1	50.7 ± 2.1	50.6 ± 2.1
WAZ at birth	629	−0.55 ± 0.9	−0.64 ± 0.9	−0.62 ± 0.9
LAZ at birth	751	−0.62 ± 1.0	−0.50 ± 1.0	−0.54 ± 1.0
WLZ at birth	746	−0.55 ± 1.1	−0.77 ± 1.1*	−0.70 ± 1.1
Gestational age at birth, wk	750	40.1 ± 1.4	40.2 ± 1.6	40.2 ± 1.6
Gestational age categories, *n* (%)				
<37 wk	20	7 (3.0)	13 (2.5)	20 (2.7)
37–40 wk	315	104 (44.4)	211 (40.9)	315 (42.0)
>40 wk	415	123 (52.6)	292 (56.6)	415 (55.3)
Infant season of birth categories, *n* (%)				
Wet season (June–October)	290	90 (37.8)	200 (38.6)	290 (38.4)
Dry season (November–May)	466	148 (62.2)	318 (61.4)	466 (61.6)
Infant diarrhea episodes (in the first 2 y of life), *n*	756	4.5 ± 3.8	4.2 ± 3.3	4.3 ± 3.5
Infant morbidity episodes (in the first 2 y of life), *n*	756	16.4 ± 8.7	16.3 ± 8.3	16.4 ± 8.4
Village categories, *n* (%)				
From Jali, Kantong Kunda, Keneba, or Manduar	194	43 (18.1)	151 (29.2)*	194 (25.7)
From remaining villages	562	195 (81.9)	367 (70.9)	562 (74.3)

1 Values are means ± SDs unless otherwise indicated. *Different from infants exclusively breastfed to age 6 mo, *P* ≤ 0.05. Fe-Fol, iron + folic acid; LAZ, length-for-age *z* score; MMN, multiple micronutrient; PE, protein-energy; WAZ, weight-for-age *z* score; WLZ, weight-for-length *z* score.

#### Infant feeding.

**FIGURE 2:**
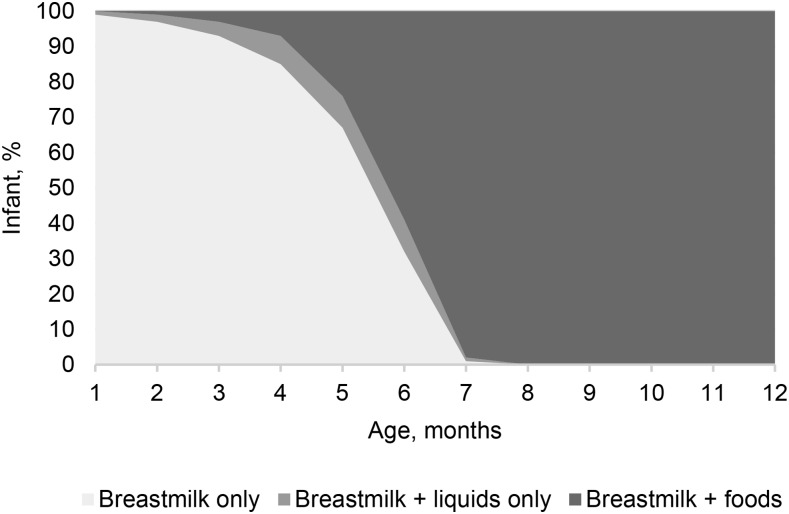
Rural Gambian infants’ feeding practice by age.

Thirty-two percent of infants were still EBF at 6 mo of age. Nine percent of infants were given breast milk and liquids only, and 59% were given breast milk accompanied by food in the first 6 mo (**Figure 2**). The mean age for introducing anything other than breast milk was 5.2 mo, resulting in 67% of infants being exclusively breastfed to 5 mo of age.

Among all infants, 1% were given water at 1 mo of age, which increased to 62% by 6 mo, and 1% were given semisolid foods at 1 mo of age, which increased to 51% by 6 mo. Other non–breast-milk foods introduced to infants <6 mo of age included sugar water (in 2% of infants), tea (4%), cow milk (3%), tinned milk (2%), powdered milk (5%), prepared weaning foods (4%), and solid foods (5%), although these foods were given on few occasions. No infant formula was given at any time point. All of the infants received some breast milk at 1 y of age, and 49% of infants were still breastfed at 2 y of age.

#### Infant feeding and postnatal growth.

**FIGURE 3:**
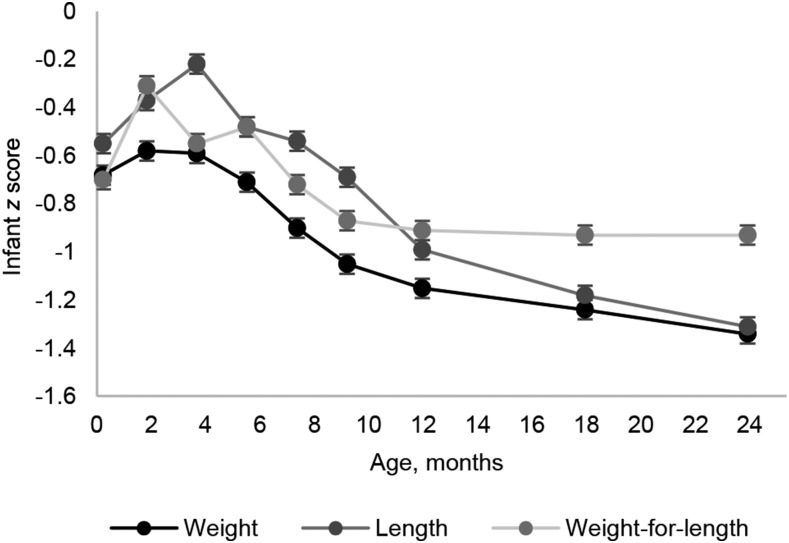
Rural Gambian infants’ anthropometric measurements by age. Values are means ± SEs.

Infants were born with low mean WAZ, LAZ, and WLZ of −0.62 ± 0.9, −0.54 ± 1.0, and −0.70 ± 1.1, respectively (Table 1); and substantial growth faltering was indicated in the first 2 y of life (**Figure 3**). Rapid growth was observed in the first few weeks after birth; however, growth faltering started at ∼3.5 mo of age (Figure 3). WAZ, LAZ, and WLZ declined to a mean ± SD of −1.34 ± 0.9 WAZ, −1.31 ± 1.0 LAZ, and −0.93 ± 0.9 WLZ at 2 y of age, by which time 26% of infants were stunted (LAZ < −2), 12% were wasted (WLZ < −2), and 23% were underweight (WAZ <−2).

**FIGURE 4:**
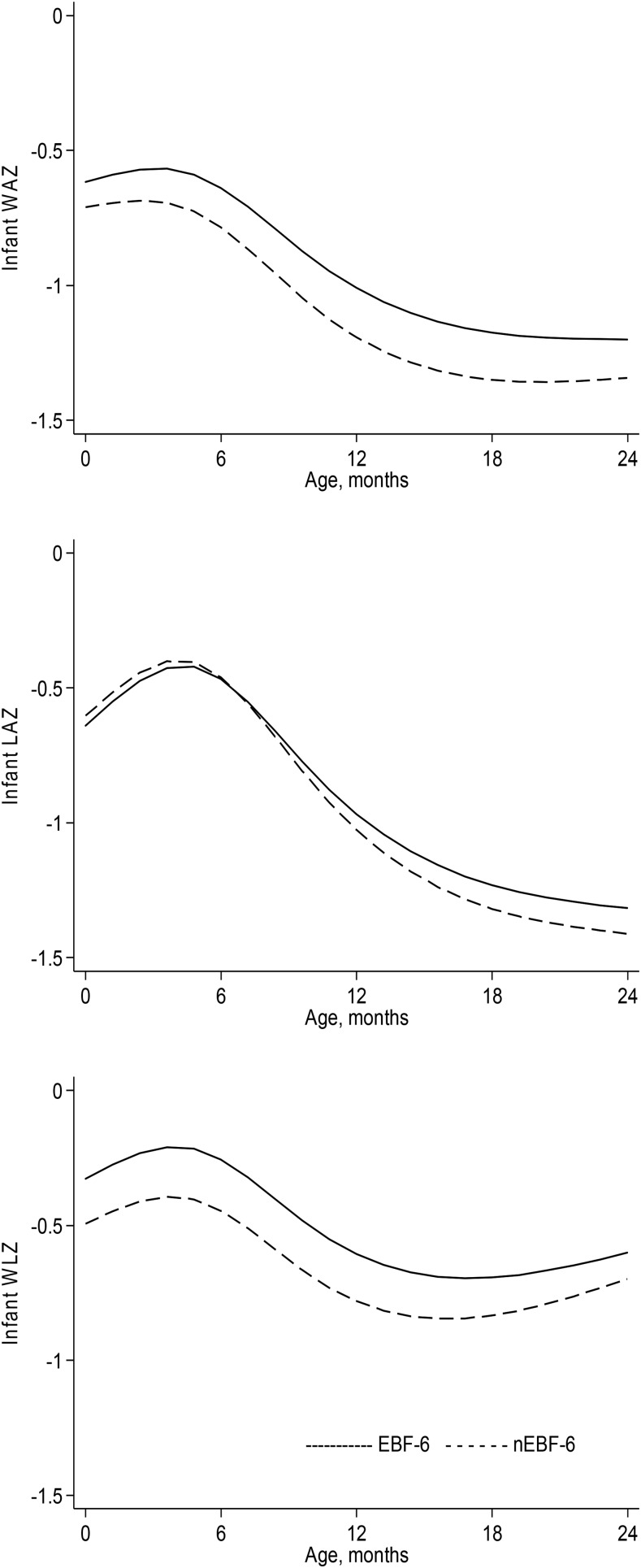
WAZ, LAZ, and WLZ trajectories between birth and 24 mo of age according to infant feeding practice. Trajectories are estimated from the multilevel models presented in Supplemental Table 2 and were adjusted for sex (female: referent), gestational age at birth, infant morbidity (no incidence), maternal height, BMI, parity (primiparous), and village (Jali, Kantong Kunda, Keneba, or Manduar), when applicable. EBF-6, infants exclusively breastfed to age 6 mo; LAZ, length-for-age *z* score; nEBF-6, infants not exclusively breastfed to age 6 mo; WAZ, weight-for-age *z* score; WLZ, weight-for-length *z* score.

Modeling infant *z* scores in the first 2 y according to infant feeding practice and adjusting for potential confounders and competing effects showed limited evidence for a difference in growth between EBF-6 and nEBF-6 infants (**Figure 4**). We found weak evidence to suggest that EBF-6 infants had a higher mean WAZ, by 0.147 *z* scores, than did nEBF-6 infants at 6 mo of age (95% CI: −0.001, 0.293 WAZs; *P* = 0.05), and for WLZ the observed difference between the groups was 0.189 (95% CI: 0.038, 0.341 WLZs; *P* = 0.01). At 6 mo of age, there was no difference in LAZ between EBF-6 and nEBF-6 infants (**Table 2**).

**TABLE 2:** Associations of exclusive breastfeeding to age 6 mo with infant growth at different ages

	Weight-for-age *z* score^1^			Length-for-age *z* score^2^			Weight-for-length *z* score^1^		
	Estimate^3^	95% CI	*P*	Estimate^3^	95% CI	*P*	Estimate^3^	95% CI	*P*
At birth	0.093	−0.040, 0.226	0.2	−0.037	−0.187, 0.114	0.6	0.166	−0.008, 0.340	0.06
At age 6 mo	0.147	−0.001, 0.293	0.05	−0.006	−0.137, 0.125	0.9	0.189	0.038, 0.341	0.01
At age 12 mo	0.183	0.011, 0.354	0.04	0.059	−0.085, 0.203	0.4	0.175	0.005, 0.345	0.04
At age 24 mo	0.143	−0.002, 0.283	0.05	0.095	−0.047, 0.237	0.2	0.097	−0.047, 0.242	0.2

1 Adjusted for sex, village, infant morbidity, gestational age at birth, parity, maternal height, and BMI.

2 Adjusted for sex, infant morbidity, gestational age at birth, parity, maternal height, and BMI.

3 The estimates show the difference in *z* score between infants who were exclusively breastfed to age 6 mo and infants who were not exclusively breastfed to age 6 mo.

Investigating the long-term influence of EBF to age 6 mo, we found weak evidence for a difference in mean WAZ between the 2 groups at 1 y of age, at which time EBF-6 infants were marginally heavier (a difference of 0.183 WAZs; 95% CI: 0.011, 0.354; *P* = 0.04) than nEBF-6 infants. A similar result was seen for WLZ, with a difference of 0.189 *z* scores (95% CI: 0.005, 0.345 WLZs; *P* = 0.04) between the 2 groups at 1 y of age. By 2 y of age, the difference in WAZ declined to 0.143 (95% CI: −0.002, 0.283 WAZs; *P* = 0.05) and for WLZ to 0.097 (95% CI: −0.047, 0.242 WLZs; *P* = 0.2). For LAZ, we found no difference between EBF-6 and nEBF-6 infants at any time point (Table 2).

Our multilevel models further showed no evidence for an interaction between infant feeding practice and the spline age terms, because infant feeding status was not associated with age-related increases (slopes) in any of the outcomes. Seasonality had a large, significant impact on infant *z* scores, and morbidity incidence had a negative influence on infant WAZ and WLZ (**Supplemental Table 2**). As a sensitivity analysis, we also modeled growth from ages 6 to 24 mo according to infant feeding practice—hence, after infant feeding practice was ascertained. With this analysis, we observed results very similar to our initial analysis, adding strength to our main findings (data not shown).

The difference in mean age at discontinuation of EBF between the EBF-6 and nENF-6 groups was small (6.2 compared with 4.7 mo), and we therefore conducted an MLM analysis post hoc with a different exposure variable: EBF to 6 mo (± 0.5 mo) compared with EBF to 3–4 mo (± 0.5 mo) with continued mixed feeding (introduction of complementary liquids or solid foods). With the use of this new categorization of infant feeding practice, the analysis showed no difference in LAZ between the 2 groups at any time point. For WAZ and WLZ, there was a difference between the 2 groups at 6 mo of age, with infants who were EBF to 6 mo having a higher mean WAZ (by 0.216; 95% CI: 0.027, 0.404 WAZs; *P* = 0.03) and mean WLZ (by 0.289; 0.093, 0.485 WLZs; *P* = 0.004) than infants who were EBF to 3–4 mo (**Supplemental Table 3**). However, these differences disappeared at 1 and 2 y of age, suggesting no long-term benefit of EBF at 6 mo of age on growth. Only 502 infants were included in this analysis, with 130 (26%) being exclusively breastfed to age 3–4 mo.

We further explored the observed difference in WLZ at birth according to feeding practice (Table 1, Figure 4). It was not possible to explain this difference by environmental, maternal, or infant characteristics; and we therefore tested the possibility of reverse causality. The method proposed by Vail et al. (26) was applied, including infants who were still EBF at 3 mo of age, testing whether their mean weight, length, and *z* score changes between 2 wk and 3 mo of age were associated with subsequent age of discontinuation of EBF. The same analysis was conducted in infants who were still EBF at 4 mo of age. We did not find that growth, either poor or good, in the first 3 or 4 mo of life was associated with when a mother stopped EBF (**Supplemental Table 4**). The method proposed by Kramer et al. (27) was also applied, and it was found that a higher mean WLZ at 3 mo of age was associated with a subsequent higher mean age at discontinuation of EBF (coefficient: 0.060; 95% CI: 0.008, 0.120; *P* = 0.03). No evidence for an association was found by using WLZ at 4 mo of age or with the use of other growth outcomes.

## Discussion

Among rural Gambian infants in this study, 32% were exclusively breastfed to 6 mo of age, and 67% to 5 mo of age, which vastly exceeded estimates from other West African countries (28). However, despite impressive EBF practices, substantial growth faltering was observed before 6 mo of age in this population. Low infant mean WAZ, LAZ, and WLZ at 2 y of age were found in this study population, which is in line with previous findings in this rural Gambian setting (5) and similar to what has been found in other West African populations (29). Furthermore, the differences in *z* scores between EBF-6 and nEBF-6 infants were small in magnitude (<0.2 SDs) across all time points in the first 2 y of life, which is far from a difference of >0.67 SDs, which corresponds to crossing a major centile band (30).

To date, to our knowledge, few published trials have investigated how following the WHO recommendation of EBF to 6 mo of age benefits growth in a low-income setting. We found 2 controlled trials from Honduras (13, 14), in which mothers were randomly assigned at 4 mo postpartum to either continue EBF to 6 mo or to feed solid foods from 4 to 6 mo and to continue breastfeeding. These data were re-analyzed by Kramer and Kakuma (3), who found that EBF to age 6 mo compared with EBF to age 4 mo did not improve infant weight or length at 6 mo of age and subsequently (ages 6–12 mo). Similar results were found in observational studies (18, 19). Kramer and Kakuma (3) further re-analyzed growth data from 3 studies, 2 from Honduras and 1 from Senegal (2 trials and 1 observational study) (13, 14, 18), and found nonsignificant higher mean WAZ, LAZ, and WLZ in infants at 6 mo of age who were exclusively breastfed to age 6 mo compared with to age 4 mo. The data presented in this current analysis add to the existing evidence of limited benefit of EBF to 6 mo of age on growth in low-income settings.

In a post hoc analysis of our data, infants who were exclusively breastfed to age 6 mo had a higher mean WAZ and WLZ at 6 mo of age than did infants who were exclusively breastfed to age 3–4 mo with continued mixed feeding. This difference, however, disappeared by 1 y of age. Changing the exposure variable meant that a large proportion of infants (36%) were excluded from the analysis, because they fell outside the new infant feeding categorization.

An interesting observation is the difference in WLZ at birth according to infant feeding practice. This observation could reflect a consequence of some maternal, environmental, or infant characteristic that determined both feeding practice and infant size at birth. However, a detailed analysis of predictors did not support this assumption. Second, this observation could reflect reverse causality, with mothers in this community choosing to continue EBF if their infant was growing well. Research from the United Kingdom has shown that rapid weight gain between birth and 3 mo of age predicted subsequent earlier age of weaning (26). Kramer et al. (27) found contrary results: a low WAZ (<−1) at 1 mo increased the risk of both weaning and discontinuation of EBF by 2 mo of age in a Belarusian population. We did not find evidence that weight gain or loss in the first 3 or 4 mo of life predicted discontinuation of EBF; however, a higher mean WLZ at 3 mo of age predicted subsequent higher age at discontinuation of EBF. This could suggest that infant size influences EBF practice in this setting and not vice versa. This possibility that larger infants are more likely to remain exclusively breastfed reinforces the conclusion that EBF to age 6 mo does not yield a growth benefit.

An important strength of this study is the comprehensive growth and feeding data collected prospectively. Infant feeding data were collected weekly, which made it possible to determine feeding practice accurately according to infant age. This further allowed the use of multilevel modeling to analyze the data longitudinally. However, we acknowledge several limitations. First, this was an observational study, with well-recognized sources of potential bias. Second, substantial error in the WLZ model was observed, because WLZ combines 2 sources of error (weight and length).

There are a number of possible reasons for the lack of a strong association between early infant feeding practice and infant growth in this rural Gambian population. First, there was a low diversity in feeding behavior observed in this population. The differences in feeding practice between the 2 groups were modest and the population had a high mean age of discontinuation of EBF (5.2 mo). Second, drivers of growth faltering in this setting are potentially so powerful that EBF to age 6 mo is not sufficient to improve growth over the long term. Several combined environmental effects, such as a highly infectious disease environment, food insecurity, poor hygiene standards and practices, and low quality of complementary foods, could, in this setting, play a larger role in poor growth than EBF to age 6 mo. Furthermore, it is likely that mothers in this study population experienced growth faltering themselves in early life, increasing the risk of an intergenerational cycle of stunting (31).

What has not received much attention as a potential contributing factor to poor growth is how entering pregnancy and lactation with inadequate nutritional status might affect breast-milk composition (32). It is well established that, regardless of maternal BMI, lactating women are capable of producing sufficient volume and macronutrient concentrations to sustain infant needs (33, 34). However, limited evidence exists for breast-milk micronutrient concentrations across the duration of EBF in mothers who experience nutritional vulnerability (32). Evidence has shown that several micronutrients in breast milk are influenced by maternal nutritional status and intake (32), increasing the need to investigate if low breast-milk micronutrient concentrations could be a contributing factor to the poor growth experienced in rural Gambia and other low-income settings. In conclusion, these results suggest that EBF to age 6 mo has limited benefit to growth in rural Gambia.

## Footnotes

8 Abbreviations used: EBF, exclusive breastfeeding; EBF-6, exclusively breastfed to age 6 mo (group designation); ENID, Early Nutrition and Immune Development; LAZ, length-for-age *z* score; LMIC, low- and middle-income country; MLM, multilevel model; MRC, Medical Research Council; nEBF-6, not exclusively breastfed to age 6 mo (group designation); WAZ, weight-for-age *z* score; WLZ, weight-for-length *z* score.

## References

[1] World Health Organization. Global strategy for infant and young child feeding. Geneva (Switzerland):WHO; 2003.

[2] World Health Organization. Nutrient adequacy of exclusive breastfeeding for the term infant during the first six months of life. Geneva (Switzerland):WHO; 2002.

[3] Kramer MS, Kakuma R Optimal duration of exclusive breastfeeding. Cochrane Database Syst Rev 2012;8:CD003517. 10.1002/14651858.CD003517.pub2 PMC7154583 22895934

[4] Victora CG, de Onis M, Hallal PC, Blössner M, Shrimpton R Worldwide timing of growth faltering: revisiting implications for interventions. Pediatrics 2010;125:e473–80. 20156903 10.1542/peds.2009-1519

[5] Prentice AM, Moore SE, Fulford AJ Growth faltering in low-income countries. World Rev Nutr Diet 2013;106:90–9. 23428686 10.1159/000342563

[6] Whitehead RG, Paul AA Growth charts and the assessment of infant feeding practices in the western world and in developing countries. Early Hum Dev 1984;9:187–207. 6376066 10.1016/0378-3782(84)90031-8

[7] Waterlow JC, Thomson AM Observations on the adequacy of breast-feeding. Lancet 1979;2:238–42. 89342 10.1016/s0140-6736(79)90248-4

[8] Giugliani ER, Horta BL, Loret de Mola C, Lisboa BO, Victora CG Effect of breastfeeding promotion interventions on child growth: a systematic review and meta-analysis. Acta Paediatr 2015;104:20–9. 26361071 10.1111/apa.13160

[9] Hawley NL, Johnson W, Nu’usolia O, McGarvey ST The contribution of feeding mode to obesogenic growth trajectories in American Samoan infants. Pediatr Obes 2014;9:e1–13. 23386576 10.1111/j.2047-6310.2012.00137.x PMC3797146

[10] Victora CG, Morris SS, Barros FC, Horta BL, Weiderpass E, Tomasi E Breast-feeding and growth in Brazilian infants. Am J Clin Nutr 1998;67:452–8. 9497189 10.1093/ajcn/67.3.452

[11] Eckhardt CL, Rivera J, Adair LS, Martorell R Full breast-feeding for at least four months has differential effects on growth before and after six months of age among children in a Mexican community. J Nutr 2001;131:2304–9. 11533271 10.1093/jn/131.9.2304

[12] Kramer MS, Guo T, Platt RW, Sevkovskaya Z, Dzikovich I, Collet JP, Shapiro S, Chalmers B, Hodnett E, Vailovich I, Infant growth and health outcomes associated with 3 compared with 6 mo of exclusive breastfeeding. Am J Clin Nutr 2003;78:291–5. 12885711 10.1093/ajcn/78.2.291

[13] Dewey KG, Cohen RJ, Brown KH, Rivera LL Age of introduction of complementary foods and growth of term, low-birth-weight, breast-fed infants: a randomized intervention study in Honduras. Am J Clin Nutr 1999;69:679–86. 10197569 10.1093/ajcn/69.4.679

[14] Cohen RJ, Brown KH, Canahuati J, Rivera LL, Dewey KG Effects of age of introduction of complementary foods on infant breast milk intake, total energy intake, and growth: a randomised intervention study in Honduras. Lancet 1994;344:288–93. 7914260 10.1016/s0140-6736(94)91337-4

[15] Engebretsen IM, Jackson D, Fadnes LT, Nankabirwa V, Diallo AH, Doherty T, Lombard C, Swanvelder S, Nankunda J, Ramokolo V, Growth effects of exclusive breastfeeding promotion by peer counsellors in sub-Saharan Africa: the cluster-randomised PROMISE EBF trial. BMC Public Health 2014;14:633. 24950759 10.1186/1471-2458-14-633 PMC4082276

[16] Jakobsen MS, Sodemann M, Biai S, Nielsen J, Aaby P Promotion of exclusive breastfeeding is not likely to be cost effective in West Africa: a randomized intervention study from Guinea-Bissau. Acta Paediatr 2008;97:68–75. 18053000 10.1111/j.1651-2227.2007.00532.x

[17] Bhandari N, Bahl R, Mazumdar S, Martines J, Black RE, Bhan MK Effect of community-based promotion of exclusive breastfeeding on diarrhoeal illness and growth: a cluster randomised controlled trial. Lancet 2003;361:1418–23. 12727395 10.1016/S0140-6736(03)13134-0

[18] Simondon KB, Simondon F Age at introduction of complementary food and physical growth from 2 to 9 months in rural Senegal. Eur J Clin Nutr 1997;51:703–7. 9347292 10.1038/sj.ejcn.1600470

[19] Khadivzadeh T, Parsai S Effect of exclusive breastfeeding and complementary feeding on infant growth and morbidity. East Mediterr Health J 2004;10:289–94. 16212203

[20] Johnson W. Analytical strategies in human growth research. Am J Hum Biol 2015;27:69–83. 25070272 10.1002/ajhb.22589 PMC4309180

[21] Dominguez-Salas P, Moore SE, Cole D, da Costa KA, Cox SE, Dyer RA, Fulford AJ, Innis SM, Waterland RA, Zeisel SH, DNA methylation potential: dietary intake and blood concentrations of one-carbon metabolites and cofactors in rural African women. Am J Clin Nutr 2013;97:1217–27. 23576045 10.3945/ajcn.112.048462 PMC3652920

[22] Ceesay SM, Prentice AM, Cole TJ, Foord F, Weaver LT, Poskitt EM, Whitehead RG Effects on birth weight and perinatal mortality of maternal dietary supplements in rural Gambia: 5 year randomised controlled trial. BMJ 1997;315:786–90. 9345173 10.1136/bmj.315.7111.786 PMC2127544

[23] Moore S, Fulford A, Darboe M, Jobarteh M, Jarjou L, Prentice A A randomized trial to investigate the effects of pre-natal and infant nutritional supplementation on infant immune development in rural Gambia—the ENID trial: Early Nutrition and Immune Development. BMC Pregnancy Childbirth 2012;12:107. 23057665 10.1186/1471-2393-12-107 PMC3534399

[24] Rees CP, Hawkesworth S, Moore SE, Dondeh BL, Unger SA Factors affecting access to healthcare: an observational study of children under 5 years of age presenting to a rural Gambian Primary Healthcare Centre. PLoS One 2016;11:e0157790. 27336164 10.1371/journal.pone.0157790 PMC4919103

[25] Fulford AJ, Rayco-Solon P, Prentice AM Statistical modelling of the seasonality of preterm delivery and intrauterine growth restriction in rural Gambia. Paediatr Perinat Epidemiol 2006;20:251–9. 16629700 10.1111/j.1365-3016.2006.00714.x

[26] Vail B, Prentice P, Dunger DB, Hughes IA, Acerini CL, Ong KK Age at weaning and infant growth: primary analysis and systematic review. J Pediatr 2015;167:317–24, e1. 26073105 10.1016/j.jpeds.2015.05.003 PMC4520860

[27] Kramer MS, Moodie EE, Platt RW Infant feeding and growth: can we answer the causal question? Epidemiology 2012;23:790–4. 23038108 10.1097/EDE.0b013e31826cc0e9

[28] Agence Nationale de la Statistique et de la D#x00E9mographie (ANSD). Senegal demographic and health and multiple indicator cluster survey (EDS-MICS) 2010#x20132011. Rockville (MD):ANSD and ICF International; 2012.

[29] International Food Policy Research Institute. Nutrition subregion profile. Western Africa 2015 [Internet] [cited 2016 Aug 29]. Available from: http://ebrary.ifpri.org/utils/getfile/collection/p15738coll2/id/130138/filename/130349.pdf.

[30] Ong KK, Ahmed ML, Emmett PM, Preece MA, Dunger DB Association between postnatal catch-up growth and obesity in childhood: prospective cohort study. BMJ 2000;320:967–71. 10753147 10.1136/bmj.320.7240.967 PMC27335

[31] Prendergast AJ, Humphrey JH The stunting syndrome in developing countries. Paediatr Int Child Health 2014;34:250–65. 25310000 10.1179/2046905514Y.0000000158 PMC4232245

[32] Allen LH B vitamins in breast milk: relative importance of maternal status and intake, and effects on infant status and function. Adv Nutr 2012;3:362–9. 22585913 10.3945/an.111.001172 PMC3649471

[33] Prentice AM, Prentice A Evolutionary and environmental influences on human lactation. Proc Nutr Soc 1995;54:391–400. 8524886 10.1079/pns19950008

[34] Prentice AM, Goldberg GR, Prentice A Body mass index and lactation performance. Eur J Clin Nutr 1994;48(Suppl 3):S78–86; discussion: S86–9. 7843163

